# Editorial: Medication Safety in COVID-19 Management

**DOI:** 10.3389/fphar.2022.940307

**Published:** 2022-07-08

**Authors:** Saibal Das

**Affiliations:** Indian Council of Medical Research – Centre for Ageing and Mental Health, Kolkata, India

**Keywords:** COVID-19, pharmacotherapeutics, medication, safety, drug development

The Coronavirus Disease-2019 (COVID-19) pandemic has brought all healthcare facilities across the globe to their limits. A plethora of therapeutic options have been tried; whilst some are evidence-based, some are not (European Medicines Agency, 2022; United States Food and Drugs Administration, 2022; World Health Organization, 2022). A few novel drugs have been launched, although most of the drugs, including combination therapies, used in COVID-19 management are repurposed ones. Some drugs have received emergency use authorizations and are lacking robust safety data. Optimizing the management of medications during the COVID-19 pandemic has become a priority. It is also pertinent to evaluate the safety profile of all these drugs on a large scale (Penman et al., 2020; Alam et al., 2021). This Research Topic has been dedicated to the safety aspects of pharmacotherapy of COVID-19.

Bailey et al. systematically reviewed the literature on clinical outcomes in metformin-treated subjects with COVID-19. The pleiotropic effects of metformin were associated with lower cardiovascular risk. Pre-existing metformin treatment offers potentially beneficial effects in patients with less severe COVID-19 infection. However, a higher risk of acidosis and lactic acidosis in patients with severe COVID-19 disease warrants that metformin should be withdrawn in patients with hypoxemia or acute renal disease.

Gao et al. explored the association between various drug treatments and the incidence of drug-induced liver injury in hospitalized COVID-19 patients. The results showed that COVID-19 patients who were treated with antibiotics, antifungals, and corticosteroids had a higher risk of drug-induced liver injury as compared to the non-users. Thus, clinicians should be warned about the development of drug-induced liver injury in hospitalized COVID-19 patients.

Jiaping et al. evaluated a pharmacovigilance signal for acute kidney injury following the use of common drugs in COVID-19 management, especially in patients with diabetes mellitus. The authors demonstrated the association of acute kidney injury with the usage of common drugs used in the management of COVID-19, especially remdesivir and tocilizumab, in patients with diabetes mellitus. These safety signals suggested individualized treatments for COVID-19 patients having various comorbidities.

Paidas et al. examined the involvement of water channels in the development of edema in multiple organs and their contribution to organ dysfunction in a Murine Hepatitis Virus-1 mouse model of COVID-19. The findings suggest the possible involvement of altered aquaporins and subsequent edema, likely mediated by the virus-induced inflammatory and oxidative stress response, in the pathogenesis of COVID-19. The authors highlighted the potential of a 15-amino acid synthetic peptide drug as a therapeutic option.

Wong et al. explored head-to-head clinical outcomes and complications associated with tocilizumab or baricitinib initiation among hospitalized COVID-19 patients receiving dexamethasone. It was found that among hospitalized patients with moderate-to-severe COVID-19 on background dexamethasone treatment, the initiation of tocilizumab or baricitinib had generally comparable effects on mortality, clinical improvement, laboratory markers, and adverse events. However, treatment with tocilizumab might lead to a faster viral clearance than that with baricitinib.

Xia et al. summarized the composition, clinical efficacy, and mechanisms of the National Health Commission-approved three Chinese medicines and three Chinese recipes for COVID-19 management. Although found to be effective, there is a lack of overall evidence of these medicines and recipes against COVID-19. Most of the data are based on virtual simulation or prediction and there is a need for robust clinical studies to establish the efficacy of these drugs in COVID-19.

Although expanding extremely rapidly, the field of therapies to treat COVID-19 remains in its evolving stage. In search of an effective definitive and/or supportive treatment of COVID-19, various studies have been conducted or are presently underway within a very short time (European Medicines Agency, 2022; United States Food and Drugs Administration, 2022; World Health Organization, 2022). Given that safety will continue to play a major role in therapeutic success, convincing short- and long-term safety data of all drugs used in COVID-19 management are required (International Union for Basic and Clinical Pharmacology, 2020). It is necessary to view safety concerns in the context of individual and specific phases of the disease to formulate a patient-specific clinical decision algorithm. De-prescribing unnecessary drugs, simplifying administration schedules, and monitoring requirements are essential. In the pre-clinical stage, mechanistic studies, biomarker development, and toxicokinetic modeling of COVID-19 therapeutics are required. After regulatory approval of these drugs, a pragmatic approach needs to be adopted. Randomized database studies, chart review, claims data, voluntary reporting, administrative data analyses, computer monitoring, direct care observations, and patient monitoring studies addressing the safety aspects of COVID-19 therapeutics should be encouraged.
